# Implementation of prostate cancer treatment decision aid in Michigan: a qualitative study

**DOI:** 10.1186/s43058-021-00125-w

**Published:** 2021-03-06

**Authors:** Roshan Paudel, Stephanie Ferrante, Jessica Woodford, Conrad Maitland, Eric Stockall, Thomas Maatman, Giulia I. Lane, Donna L. Berry, Anne E. Sales, James E. Montie, for the Michigan Urological Surgery Improvement Collaborative (MUSIC), Ann Arbor, Michigan, USA

**Affiliations:** 1 Department of Learning Health Sciences, University of Michigan, Ann Arbor, MI USA; 2 Michigan Urological Surgery Improvement Collaborative, University of Michigan, Ann Arbor, MI USA; 3 University of Michigan, Ann Arbor, MI USA; 4 Sherwood Medical Center, Detroit, MI USA; 5 Capital Urological Associates, Okemos, MI USA; 6 Michigan Urological Clinic, Grand Rapids, MI USA; 7 Department of Urology, University of Michigan, Ann Arbor, MI USA; 8 Department of Biobehavioral Nursing and Health Informatics, University of Washington, Seattle, WA USA

**Keywords:** Shared decision-making, Decision aid, Prostate cancer treatment

## Abstract

**Background:**

The American Urological Association White Paper on Implementation of Shared Decision Making (SDM) into Urological Practice suggested SDM represents the state of the art in counseling for patients who are faced with difficult or uncertain medical decisions. The Michigan Urological Surgery Improvement Collaborative (MUSIC) implemented a decision aid, Personal Patient Profile-Prostate (P3P), in 2018 to help newly diagnosed prostate cancer patients make shared decisions with their clinicians. We conducted a qualitative study to assess statewide implementation of P3P throughout MUSIC.

**Methods:**

We recruited urologists and staff from 17 MUSIC practices (8 implementation and 9 comparator practices) to understand how practices engaged patients on treatment discussions and to assess facilitators and barriers to implementing P3P. Interview guides were developed based on the Tailored Interventions for Chronic Disease (TICD) Framework. Interviews were transcribed for analysis and coded independently by two investigators in NVivo, PRO 12. Additionally, quantitative program data were integrated into thematic analyses.

**Results:**

We interviewed 15 urologists and 11 staff from 16 practices. Thematic analysis of interview transcripts indicated three key themes including the following: (i) P3P is compatible as a SDM tool as over 80% of implementation urologists asked patients to complete the P3P questionnaire routinely and used P3P reports during treatment discussions; (ii) patient receptivity was demonstrated by 370 (50%) of newly diagnosed patients (*n* = 737) from 8 practices enrolled in P3P with 78% completion rate, which accounts for 39% of all newly diagnosed patients in these practices; and (iii) urologists’ attitudes towards SDM varied. Over a third of urologists stated they did not rely on a decision aid. Comparator practices indicated habit, inertia, or concerns about clinic flow as reasons for not adopting P3P and some were unconvinced a decision aid is needed in their practice.

**Conclusion:**

Urologists and staff affiliated with MUSIC implementation sites indicated that P3P focuses the treatment discussion on items that are important to patients. Experiences of implementation practices indicate that once initiated, there were no negative effects on clinic flow and urologists indicated P3P saves time during patient counseling, as patients were better prepared for focused discussions. Lack of awareness, personal habits, and inertia are reasons for not implementing P3P among the comparator practices.

**Supplementary Information:**

The online version contains supplementary material available at 10.1186/s43058-021-00125-w.

Contributions to the literature

1. There are documented gaps in integrating shared decision-making into clinical care, and implementation of decision aids in routine clinical practice is fraught with operational challenges.
2. Our study adds to the implementation science literature as we assessed how diverse urology practices were implementing a treatment decision aid, previously evaluated in randomized trials. Our study assesses why some urology practices are more inclined to implement decision aids while also pointing out operational concerns about decision aids.
3. Our findings provide compelling evidence about the significance of local context in implementing interventions and reinforce the importance of implementation science to assess adoption, acceptability, and appropriateness of decision aids.

## Background

Treatment for early-stage favorable-risk prostate cancer is sensitive to the preferences of patients and often the providers. There is an increased emphasis on the shared decision-making (SDM) process to arrive at a decision made jointly by the patient and his clinician(s) based on the patient’s values, preferences, and treatment goals. SDM is a complicated and nuanced process in medicine. An effective SDM process requires an informed patient being engaged in their treatment decision and clinicians being responsive to patients’ values, preferences, and treatment goals [1]. Evidence points to clinicians potentially not being as sensitive to patient’s preferences as needed and using their own biases and interests to influence the decision-making process. For example, Hoffman et al. found that patients whose diagnosis was made by urologists who treated prostate cancer were more likely to receive upfront treatment, including radical prostatectomy, cryotherapy, or brachytherapy performed by their urologist [2].

A growing body of evidence points to the use of decision aids to guide the shared decision-making process [3]. Decision aids for men with localized prostate cancer (LPC) have shown promise in minimizing decisional conflict and regrets, increasing decisional satisfaction and overall satisfaction with treatment outcomes [4–6]. Decision aids have also shown to reduce the number of patients choosing invasive surgical procedures in favor of more conservative options [5].

Existing clinical guidelines encourage clinicians to ask about the values, preferences, and treatment goals of their patients, provide unbiased information to help patients make informed decisions and engage their colleagues in other specialties to participate in the interdisciplinary process of helping patients make the right choices [7, 8]. Additionally, experts recommend that clinicians respect the level of desired involvement from patients as evidence also points to varying level of preferences for engagement [9]. For example, some patients would like to be actively involved in decisions about their treatment options, while others would likely defer to their physicians’ expertise [10, 11].

There are documented gaps in integrating SDM into clinical care and many of the tools developed to support SDM process are not adequately used or sufficiently compatible with routine clinical practice [12, 13]. The Michigan Urological Surgery Improvement Collaborative (MUSIC) established in 2011, a physician-led quality improvement program, comprised of a consortium of 46 urology practices and more than 250 urologists, collectively representing 90% of the urologists in the state, is focused on improving urologic care in Michigan. MUSIC’s data collection and analysis infrastructure is supported by the Blue Cross and Blue Shield of Michigan [13].

A large umbrella organization such as MUSIC has the potential to increase adoption of SDM in their practices and test decision aids that have been developed and validated for wider implementation. MUSIC began implementing a decision aid tool called Personal Patient Profile-Prostate (P3P) in early 2018 to help newly diagnosed prostate cancer patients make an informed and shared decision with their clinicians about their prostate cancer treatment options. The P3P decision aid has been shown to be efficacious in reducing decisional conflict in two multi-center, randomized trials [4, 14].

The P3P decision aid tool asks men about their socio-demographic status and a set of personal factors known to impact decision making, recent urologic health history, how much the users wants to participate in the decision, and the status of their decision [15, 16]. P3P includes questions from the shortened version of Expanded Prostate Cancer Index Composite (EPIC), known as EPIC-26. Patients complete the one-time, online questionnaire at home or on the day of their appointment that captures their bowel, urinary, sexual, and hormonal functions in the past 4 weeks using the EPIC-26. After patients complete the questionnaire, the intervention component provides information on prostate cancer treatment options tailored to men’s own preferences along with testimonials from previous patients who may have chosen either active treatment or surveillance as options. The video clips coach the user on how to communicate personal preferences and values to their clinicians. A one-page summary report is generated for the patient and their provider; this is electronically made available in the MUSIC Registry for the clinic staff to provide to the patient’s provider. The report is intended to guide treatment discussions by better understanding the patient’s main priorities and concerns. Patients who elect surgery as their treatment are enrolled into the MUSIC PRO program and P3P responses become PRO baseline responses, as long as P3P was completed within 6 months of surgery.

### Study purpose

The purpose of this study was to perform a qualitative assessment of P3P implementation at participating MUSIC urology sites, to understand how urology practices that implemented P3P integrated the program into their workflow. We also wanted to better understand reasons why practices that have not yet implemented P3P were reluctant to implement it.

### Aims

The primary aim was to assess facilitators and barriers to successful implementation of P3P into local clinics. We sought to understand the determinants of P3P implementation based on the experiences of eight participating urology clinics (implementation practices) and to assess perspectives from nine practices that had not yet implemented P3P (comparator practices). Additionally, our secondary aim was to understand both urologists and clinic staffs’ perspectives about P3P and SDM in general.

## Methods

We interviewed urologists and clinic staff including nurses, medical assistants, and practice administrators from P3P implementation and comparator practices between the months of August to October 2019. Implementation practices were defined as practices where at least one urologist had enrolled newly diagnosed prostate cancer patients in P3P by the time of the interview date. Comparator practices were defined as urology groups affiliated with MUSIC that had not enrolled any patients in P3P at the time of the interview date.

Through interviews, we sought to explore the following key questions:

- How have implementation sites integrated P3P into normal clinic flow and how have urologists used P3P reports in clinical encounters? How P3P might have changed the way urologists were having treatment discussions with their patients?
- How have urology clinic staff and urologists perceived their patients’ acceptance of P3P and what feedback have they received from patients who have completed P3P?
- How familiar were the comparator sites with P3P and what were reasons they had not considered implementing P3P in their practices?

### Data collection

Interview instruments were developed for clinical and non-clinical staff based on the Tailored Interventions for Chronic Disease (TICD) framework. The TICD framework was developed to contribute knowledge on how to improve healthcare for patients with chronic diseases [17]. The TICD framework was selected for its emphasis on tailoring and developing interventions to determinants of practice in chronic illness care [18]. TICD has been used in urologic services [19] and integrates elements of other frameworks including the Consolidated Framework for Implementation Research (CFIR) and the Theoretical Domains Framework (TDF). Flottorp et. al. developed the TICD checklist to identify the most important sets of determinants of practice for tailoring and reporting interventions [20]. The determinants of practice are facilitators and barriers that enable or prevent improvements in clinical settings. We used the TICD checklist with 57 potential determinants of practice grouped in 7 domains to develop our interview instruments and codebook. Our interview questions were primarily derived from domains including guideline factors, individual health professional factors, patient factors, and professional interactions. Interview guides were prepared by RP, reviewed and edited by the study team including JM, SF, and DB. Participants were recruited with a purposive sampling method by study team members (JM and SF) and included all practices implementing and not implementing P3P. Requests to participate in interviews were emailed by JM and SF. Participants were told that the interviewer is a doctoral student at the University of Michigan who was interested in assessing the facilitators and barriers to P3P implementation. Interviewer then followed up with participants to schedule phone interviews.

Semi-structured phone interviews were conducted between the months of August to October 2019 by a team member (RP). Interview time with clinicians ranged from 9 to 21 min with an average interview time of 14 min. Interviews with staff were held for 6 to 28 min with an average interview time of 13 min. All phone interviews included only the interviewer and the interviewee except for two interviews that included a staff-clinician dyad and staff-staff dyad. Interviews were recorded, with permission, and transcribed for analysis. Audio recordings were automatically transcribed with TRINT (Trint Ltd, London, UK), a web-based automated transcription software. Each automated transcript was then manually audited for accuracy and edited if transcription errors were found.

### Data analysis

Interviews were coded deductively into the TICD framework using template analysis. We used NVivo 12 (QSR International, Sydney, Australia), qualitative data analysis software for managing the transcription data and tracking the codes. Two study team members (RP and JW) independently coded the interview transcripts. Coding meetings were held periodically to compare codes and discuss emerging themes. Disagreements in coding and discrepancies in codes were resolved through discussions aiming to arrive at consensus.

## Results

We invited 31 individuals from 18 practices to participate in the semi-structured interviews and 26 individuals representing 16 practices agreed to be interviewed. Out of 26 interviewed, 15 were urologists and 11 were urology clinic staff from 8 implementation and 8 comparator practices. Characteristics of the eight implementation and eight comparator practices are presented in Table 1. In general, the mean number of prostate cancer cases in 2019 was 251 among implementation practices, and 451 among comparator practices.

**Table 1:** Characteristics of implementation and comparator urology practices

	Implementation Practices (*n* = 8)	Comparator Practices (*n* =8)
Prostate cancer cases (2019), mean (SD)	251 (330)	451 (556)
Number of urologists interviewed, *n* (%)	10 (66%)	5 (33%)
Number of urology clinic staff interviewed, *n* (%)	6 (55%)	5 (45%)

### Key Themes

Interviews were coded to 4 out of 7 relevant TICD domains including (i) guideline factors, (ii) individual health professional factors, (iii) patient factors, and (iv) professional interactions. Findings are grouped into 6 key themes, presented below:

#### Theme 1: Overall positive impression of P3P

To assess overall impression of P3P, we asked clinicians and staff from implementation practices the following questions:

A. How would you describe your experience implementing P3P in your practice?
B. How do you use the P3P report in clinical encounters?
C. How has P3P changed the way you have treatment discussions with your patients?
D. What impact has P3P had, if any, on clinic time/workflow?

Overall, among all urologists who implemented P3P, over 80% asked their patients to complete the P3P questionnaire routinely, and used the one-page report during the treatment discussion. About 70% of the urologists interviewed had an overall positive impression of P3P and staff were also overwhelmingly positive about P3P as well. This representative quote highlights one urologist’s impression of P3P:

P3P helps patients to know that we have taken interest in their concerns. Previously we probably glazed over their concerns and talked about things like Gleason score or 10-year outcomes. P3P focuses on quality of life, which is important to patients. P3P shifts focus of conversation from what we want to talk about to what *they* want to talk about. (Urologist, Implementation Practice)

In addition to having positive impressions of P3P, over half of the implementation site urologists interviewed indicated that P3P saves time and did not require much effort.

P3P has zeroed, the number of times I get those additional phone calls a day later from the spouse or the patient saying, ‘I didn’t catch all that, can you go over it again on the phone.’ (Urologist, Implementation Practice)

….if they [urology practices] implement this program [P3P], they are going to find, it saves them time. People might think that, or practices might think that it’s time consuming. It’s really not. (Urologist, Implementation Practice)

Even though three quarters of urologists interviewed had an overall positive impression of P3P, some remained skeptical about P3P’s impact on patients’ decision-making. Over a third of urologists felt they give patients enough information with or without a decision aid and P3P has not changed how they discuss treatment options, highlighted by the following clinician feedback:

A lot of the answers I get, I actually get just by talking to the patient and discussing the pros and cons of various treatment options. (Urologist, Implementation Practice)

It’s important to look at if patients are better prepared because of P3P or are engaged patient[s] more likely to complete P3P? Would these patients come in more prepared even if P3P wasn’t available? (Urologist, Implementation Practice)

#### Theme 2: P3P is compatible with clinic workflow

Clinicians and staff also reported that P3P does not disrupt busy clinic workflow. Some practices have made P3P part of the *standard ask* and routinely ask patients to complete P3P prior to treatment discussion conversations with their urologists. While some urologists reported using information on P3P to help guide discussions and answer questions from patients or their family members, other urologists glanced at P3P immediately before a patient visit to get “a general sense of where the patient is in terms of voiding and sexual functions” (an implementation site urologist).

Urologists also reported using P3P as a way to clarify patients’ thought processes and believed that P3P provided patients with “talking points” and prepared them for their treatment discussions. The following representative quotes illustrate P3P's compatibility with clinic workflow based on experiences of multiple staff and clinicians:

P3P helps patients with talking points. Some patients have many questions so having a way to discuss them helps [the patient]. (Urologist, Implementation Practice)

…but sometimes, if I don’t really get a good snapshot of that [patient’s preferences] or the patient is all over the place mentally., I would tell them that this tool [P3P] may help. (Urologist, Implementation Practice)

Implementation urologists and staff also indicated that P3P provides an opportunity for practices to improve patient-reported outcomes’ (PRO) process adoption and enrollment, measuring a patient’s functional status and health quality before surgery and at several time points after.

I think it’s a good initial step to get the patient in the system and... If they do have surgery, they will be already in the system for PRO…. (Staff, Implementation Practice)

I think P3P and PRO go hand in hand. I think they are both great sources of information.... (Staff, Implementation Practice)

#### Theme 3: patient receptivity is high

Urologists and clinic staff within implementation practices indicated high patient receptivity when patients were requested to complete P3P prior to treatment discussion appointments. This was quantitatively confirmed, as analysis of P3P data indicated that half (*n*=370) of the newly diagnosed patients (*n*=737) were enrolled in P3P with 78% completion rate, which accounts for 39% of all newly diagnosed patients in these practices.

[Experience] it’s been very good, overall. We use it [P3P] for every patient we diagnose with prostate cancer... [T]he P3P becomes an important aspect of what we do in terms of gleaning information or to improve the care that we render to them [patients]. And in that regard, I’ve had no patients, so far, refused to participate in P3P. (Urologist, Implementation Practice)

The positive aspect would be several patients have said that it kind of focused their mind on the task ahead and the task being they are doing to make a decision. (Urologist, Implementation Practice)

Patients are receptive. Once patients are asked they become very willing to complete P3P because they look at it as a part of their care. (Staff, Implementation Practice)

Despite a high level of positive patient receptivity reported by implementation sites’ clinicians and staff, other clinicians and staff reported patient-level barriers to completing P3P including access to technology and perceived intrusive nature of some P3P questions.

Patients who are not computer savvy and patients without emails are resistant. Those without support systems are also unlikely to complete P3P. (Staff, Implementation Practice)

Most patients are completing the questionnaire [P3P]. Most patients are neutral. Some patients complain about it, they feel it’s [P3P] intrusive. (Urologist, Implementation Practice)

…And when I asked them [patients] at that point how they felt about the tool [P3P], they give responses [that] are not uniformly positive. Some people will say, I didn’t really understand why they are asking me these personal questions. They didn’t, maybe even know, that the range of topics was what we were going to talk about. (Urologist, Implementation Practice)

#### Theme 4: P3P focuses on what is important to patients

The goal of enhancing patient experience was the primary reason for P3P implementation, as indicated in interviews with urologists and clinic staff among implementation sites. These key informants reported that P3P focused treatment discussions on items that are important to patients. Urologists who have implemented P3P used the one-page report as a way to capture the patients’ views on what is important to the patient. Other urologists have used the report just before seeing patients to get a “general sense of where they are” while others are using P3P to help patients formulate questions or to guide the treatment decision-making process.

P3P helps patients to know that we have taken interest in their concerns…P3P shifts focus of conversation from what we [physicians] want to talk about to what they [patients] want to talk about. (Urologist, Implementation Practice)

[I] like that it [P3P] objectively captures the patient’s views on what’s important. (Urologist, Implementation Practice)

A diagnosis is so overwhelming for patients and the whole family, this [P3P] has been very good for the patients to be able to start wrapping their head around what they are going to be hearing and talking about. (Staff, Implementation Practice)

P3P helps patients with talking points. Some patients have many questions so having a way to discuss them helps them. (Urologist, Implementation Practice)

#### Theme 5: Clinician’s attitudes towards SDM varied

Implementation and comparator site clinicians had mixed attitudes towards SDM. About 40% of urologists who implemented P3P felt they gave patients enough information with or without a decision aid and that P3P did not change how they discussed treatment options with patients without eliciting patients’ values, preferences, and treatment goals. Some implementation site urologists, despite accepting the importance of SDM in treatment discussions, stated that they did not rely on P3P for SDM. For instance, academic urologists we interviewed employed a nuanced approach to SDM than those who practiced in community settings. These urologists generally saw informed patients who wanted second opinions or were aware of treatment options. When patients came in for second opinions, academic urologists discussed outcomes but did not engage in the shared decision-making process. Additionally, they reported rarely looking at P3P reports, even though their patients may have completed them.

Most of my patients come to me for a second opinion. So, they have already seen a urologist, they have already seen a medical oncologist or radiation oncologist. So they already have a pretty good understanding of what their disease is. [But] for the people I see the first time after their diagnosis, before seeing other providers, they get a packet and my guess is that they get the P3P tool as well. When they come to see me, we have an in-depth conversation about this disease. (Urologists, Implementation Practice)

My overall impression is that this [P3P] is probably not as valuable as I hoped it would be… When you present the different outcomes, they [patients] will end up saying what is most important to them. If I tell them that these are the side effects of radiation, these are the side effects of surgery, they are picking one [preferences], and by the nature of picking one, they are telling me what’s most valuable to them. (Urologist, Implementation Practice)

Similarly, some community urologists who saw newly diagnosed patients were more likely to ask patients to complete P3P but did not routinely engage in the shared decision-making process.

Most patients are happy to see [the P3P] results [report] … They get the print-out and they get the guided view of things. But then again, I do have a lot of less sophisticated patients and some of them don’t find the tool [P3P] effective. Some people just want me to tell them what to do. (Urologist, Implementation Practice)

…After the biopsy is reviewed, they [patients] are then given informational pamphlets and booklets on treatment options and start the process to implement P3P. Then they come back typically in two weeks for a one-hour consultation to discuss treatment options. Then I use the information on P3P to help guide my discussion and answer any questions that they might have.

For these urologists, P3P had not changed the way they discussed treatment options with their patients. They usually provided patients with options, answered questions while not fully engaging in SDM. About a third of the urologists have questions about the overall impact of P3P on patient’s decision-making. They expressed skepticism about decision aids as these representative quotes highlight:

A lot of the answers I get, I actually get just by talking to the patient and discussing the pros and cons of various treatment options. (Urologist, Implementation Practice)

I already know the patient from several encounters at that point and I kind of have a feeling of where they want to go. So, I feel like the extra, I am not saying in every case, but in some cases, the burden of having them [patients] set an extra 15 minutes to fill out the form or online module becomes a little added burden on the patient especially after I just gave them a diagnosis of cancer…It’s just an extra burden when I already have a lot of the information in my mind with the patients. (Urologist, Implementation Practice)

#### Theme 6: Lack of awareness, personal habits, and inertia are reasons for non-implementation

Comparator site urologists indicated lack of awareness, personal habit, or organizational inertia as reasons for not using P3P. Many of the urologists interviewed were less familiar with P3P as a decision aid. Some reported having heard of P3P at MUSIC tri-annual collaborative-wide meetings, however, had not engaged in the P3P project prior to the interview. The urologists who indicated personal habit or organizational inertia as reasons for not implementing P3P suggested that they were not necessarily opposed to P3P, but saw little value in implementing this decision aid tool. Others indicated that decision aids, like P3P, could go against urologists’ natural flow of counseling these patients and were opposed to implementing P3P.

The issue we have is… it’s just a matter of starting up the program. Once the program is started and we get past introducing it to the staff and how to implement it within the group. It’s not a big deal. It’s just a matter of getting past that initial phase. (Urologist, Comparator Practice)

Staff from comparator sites were primarily concerned about the impact of provider workflow preferences on P3P implementation, specifically as it relates to multi-provider practices without standardized workflow. They seemed uncertain about operationalizing P3P in the context of wide variations in how urologists within a large multi-site/provider practice prefer to interact with patients, as illustrated by this representative quote:

…but they all [urologists] have a little bit different practice in how they, you know, have the discussion with their patient once they’ve been diagnosed with prostate cancer. Some of them do it in person, some of them do it over the telephone and then follow up, you know, after a phone conversation, they have them come into the office but some of them don’t discuss anything until they come into the office. So, I think that will be maybe a little bit challenging for the office staff and how it’s implemented, depending on who the provider is. (Staff, Comparator Practice)

Some comparator urologists were not convinced that a decision aid was needed in their practice, suggesting patients defer the decision-making to their urologist(s) and that the clinician provide enough information to their patients so a decision aid was not needed in their practice. Notably, no comparator urologist acknowledged or addressed the need to hear the patient-reported personal values or preferences when discussing the treatment decision.

…certainly, there are patients that don’t want to hear anymore. They just want you to make their decisions. But I think most of my patients come in typically with a spouse and then not uncommonly they go home and talk to the rest of the family members to make the decisions, you know? (Urologist, Comparator Practice)

And, obviously about prostate cancer my canned talk has changed over the years. But, I basically tried to hit on every topic and, you know, you modify it based on person’s state of health or their age or their fear level or their, you know, the PSA or the Gleason score… But in my one hour [with them] I think I tell people the same thing three times. You know I think I overkill in the instruction process. (Urologist, Comparator Practice)

Table 2 shows facilitators and barriers to P3P implementation organized by domains from the TICD framework. The most common facilitators included P3P’s operational simplicity and the most common barriers were skepticism about shared decision-making and concerns about clinical workflow. Urologists, who have been in practice for a while with established routine, perceived P3P would add work and interfere with their long-standing workflow. Additionally, patient access to the Internet, e-mail, or computers to complete P3P was identified as barriers by clinic staff. Organizational factors that deterred P3P implementation at comparator sites included buy-in from clinic leadership, clinicians and staff. Administrative staff turnover and resulting impact on organization capacity at smaller practices also hindered P3P implementation while larger practices reported organizational buy-in as one of the key barriers.

**Table 2:** Facilitators and barriers to P3P implementation

TICD determinant domains	Perceived facilitators	Perceived barriers
**Guideline factors**	• Simplified process for implementation • P3P serves as an “on-ramp” to PRO surveys	• Provider skepticism regarding SDM • Provider skepticism regarding P3P
**Patient factors**	• Enhances patient experience • Helps to glean information from patients • Objectively captures patient views • MUSIC provided tablet computers to bridge the gap in access	• Added burden on patients • Patients perceiving P3P questions to be intrusive • Patient access to email, internet and computers
**Individual provider factors**	• Helps urologists have treatment discussions • Incorporates patient input in decision-making	• Perception that P3P could go against urologists preferred workflow • P3P vs. other tools (AUA-SI, or IPSS-SF)
**Provider interactions**	• Teamwork-coordination between urologists, mid-level providers and coordinators, navigators • Creating internal notification • Coordination with MUSIC staff	• Incoming referrals for treatment (patients seen by different urologists so opportunity to complete P3P is absent)
**Capacity for organizational change**	• Clinical champions • Dedicated staff assigned to facilitate P3P implementation • Electronic Health Record integration (alerts, reminders and EHR smart phrases for physician orders)	• Organizational factors such as organizational buy-in, IRB issues • Staffing—turnovers and capacity • Competing priorities • Fear of change or fear of forgetting

## Discussion

The integration of SDM into routine clinical practice to help newly diagnosed prostate cancer patients make treatment decisions based on their values, preferences, and goals is conceptually sound but fraught with operational challenges. MUSIC has been supporting urology practices in Michigan to implement a decision aid, P3P, by simplifying and streamlining the processes, making P3P available to patients to complete at home or in clinic, and helping staff and clinicians integrate P3P into routine clinical practice. Our findings indicate that MUSIC’s initial efforts are advancing the goal of increasing the proportion of newly diagnosed prostate cancer patients who are exposed to a decision aid tool, P3P. Our findings also indicate that clinicians and staff involved in implementing P3P routinely ask their patients to complete the P3P questionnaire and use the one-page report during treatment discussions.

Overall, we found that community urologists who use P3P regularly had an overwhelmingly positive impression and once implementation has been started, staff enthusiasm seemed high. We found that despite being open to the concept of SDM, some urologists questioned the utility of decision aids on patients’ decision-making. For instance, over a third of urologists who routinely use a decision aid felt they give patients enough information with or without a decision aid, and P3P had not changed how they discuss treatment options with their patients. Many of the urologists we interviewed stated making treatment decisions on behalf of their patients. Prior qualitative studies have documented this phenomenon of patients deferring treatment decisions to their clinicians [21, 22]. Some of these urologists also felt their patients were already well-informed about the disease when they visit them for second opinions, negating the need for decision aids. Urologists at comparator sites indicated habit or inertia as reasons for not using P3P. Prior studies have shown that lack of awareness about decision aids among clinicians as the main barrier to using decision aids in routine clinical practice [23]. Lack of awareness of decision aids was not the primary reason in MUSIC practices as most of the urologists we interviewed were aware of the availability of P3P as a decision aid. However, we did find that many of the comparator site urologists were not convinced a decision aid was needed in their practice, while others indicated that decision aids could go against their natural flow of how they counseled their patients. These observations are consistent with prior studies that have documented clinician’s concerns about the use of decision aid based on the perception that clinicians were already involving patients in decisions, therefore no perceived need to change or to adopt decision support tools [24].

We identify several opportunities for further investigation including which implementation strategies are likely to encourage reluctant practices to implement decision aids, and whether P3P supports increased uptake of PROs post-surgical interventions. Additionally, it would be useful to understand how P3P impacts decisional satisfaction or decisional regret and the tolerance of side effects among those who choose active treatment versus active surveillance.

### Strengths

Our study has several strengths: first, we explored perspectives of implementation and comparator practices representing urban, suburban, and rural areas in Michigan. These practices varied based on their size including number of patients they treat as well as number of urologists they employ. Additionally, practices also varied by organization type as we included academic, private, and multi-specialty practices. In addition to interviewing urologists, we also interviewed staff partners including program coordinators, medical assistants, and nurses, who are critical to successful implementation, to gain operational and contextual data that influenced P3P implementation.

### Limitations

Limitations include the possibility that our interviewees included only those who tend to be more receptive to being interviewed. As we compiled the list of potential interviewees, we may have introduced bias. It is also possible that we may have missed urologists and staff from implementation and comparator sites who had differing options about SDM and P3P. Our learnings might not be transferable to other states because MUSIC is unique to Michigan as a statewide quality improvement collaborative.

## Conclusion

Overall, urologists and staff affiliated with implementation sites indicated that P3P focuses treatment discussion on items that are important to patients and that, once implemented, clinic patient flow is not adversely impacted. This study contributes to our understanding of the challenges facing a population-based implementation of a decision aid for prostate cancer care.

## Supplementary Information

**Additional file 1.**

## Data Availability

Not applicable. Since this is a qualitative study, participants were assured confidentiality and anonymity; hence, interview transcripts are not available for dissemination.

## Abbreviations

AUA-SI: American Urological Association—Symptom Index
CFIR: Consolidated Framework for Implementation Research
EPIC: Expanded Prostate Cancer Index Composite
IPSS: International Prostate Symptom Score
LPC: Localized prostate cancer
MUSIC: Michigan Urological Surgery Improvement Collaborative
P3P: Personal Patient Profile-Prostate (P3P)
PRO: Patient-reported outcomes
SDM: Shared decision-making
SHIM: Sexual Health Inventory for Men
TDF: Theoretical Domains Framework
TICD: Tailored Implementation in Chronic Diseases
