# Correction: Implementation of prostate cancer treatment decision aid in Michigan: a qualitative study

**DOI:** 10.1186/s43058-023-00410-w

**Published:** 2023-03-20

**Authors:** Roshan Paudel, Stephanie Ferrante, Jessica Woodford, Conrad Maitland, Eric Stockall, Thomas Maatman, Giulia I. Lane, Donna L. Berry, Anne E. Sales, James E. Montie, for the Michigan Urological Surgery Improvement Collaborative (MUSIC), Ann Arbor, Michigan, USA

**Affiliations:** 1 Department of Learning Health Sciences, University of Michigan, Ann Arbor, MI USA; 2 Michigan Urological Surgery Improvement Collaborative, University of Michigan, Ann Arbor, MI USA; 3 University of Michigan, Ann Arbor, MI USA; 4 Sherwood Medical Center, Detroit, MI USA; 5 Capital Urological Associates, Okemos, MI USA; 6 Michigan Urological Clinic, Grand Rapids, MI USA; 7 Department of Urology, University of Michigan, Ann Arbor, MI USA; 8 Department of Biobehavioral Nursing and Health Informatics, University of Washington, Seattle, WA USA

**Correction: Implement Sci Commun 2, 27 (2021)**

**https://doi.org/10.1186/s43058-021-00125-w**

Following the publication of the original article [1] the authors requested to update the “Competing interests” section as follows:

“The authors declare that Anne Sales is co-Editor-in-Chief of the journal."
