# Supplementary material for: Implementation of prostate cancer treatment decision aid in Michigan: a qualitative study
Source: Implement Sci Commun. 2021 Mar 6;2:27. doi: 10.1186/s43058-021-00125-w (PMC7936475; doi:10.1186/s43058-021-00125-w)
Supplement: Supplementary file 1 — Additional file 1. [file 43058_2021_125_MOESM1_ESM.docx]

| **Domain (Theme)** | **Parent Code** | **Child Node (if applicable)** | **Definition** | **Include** | **Do Not Include** | **Example** |
| --- | --- | --- | --- | --- | --- | --- |
| Guideline Factors | Cultural appropriateness |  | Description of whether P3P is appropriate to implement in all or specific types of societal contexts. | Societal factors (social, political, economic) that impact the ability to implement P3P in the community they work with. | Case by case examples (see patient preference). | “Quote from interview to illustrate when to use this code.” |
| Guideline Factors | Compatibility (Recommended behavior) |  | P3P compatibility with current clinical practices. | Workflow related factors such as how practices deliver biopsy results | Factors that are not related to workflow | “…I think it’s a good initial step to get the patient in the system…. If they do have their surgery they will be already in the system for PRO and all that.” |
| Guideline Factors | Effort (recommended behavior) |  | Effort (time) is required to implement P3P | Time, effort, re-work needed to sign patients up for P3P and any f/u required | Patient related efforts (we are looking at clinic level efforts) | “I have been pleased with it. It’s been seamless. It’s not a lot of effort.”  “…I think in terms of change in practice, it hasn’t put that much of a burden on that.” |
| Individual Health Professional Factors | Awareness and familiarity with P3P |  | Interviewee is familiar with P3P | Do they know about P3P? Have they been exposed to P3P? | Familiarity with other instruments | “It’s an awesome idea. I am all for it. I have been pushing to get it at my clinic.” |
| Individual Health Professional Factors | Knowledge about own practice |  | Interviewee is knowledgeable about her/his own practice in relationship to P3P or shared decision-making | Awareness of what happens within their clinic. Can they talk about how the process unfolds? | General knowledge not related to practice operations | “Well, we were actualy asking questions in the not haphazard manner, but it wasn’t something logical or progressive.”  “So, when a patient gets a phone call regarding their positive biopsy results, they are also informed that they will be receiving an email link that we want them to complete.” |
| Individual Health Professional Factors | Agreement with the recommendation (P3P use) |  | Interviewee is in agreement with P3P being used as a decision-aid | Does the interviewee agree that P3P is a good tool for patients with favorable risk prostate cancer? | Patient level issues – agree/disagree because patient related barriers | “I always look at the survey. I look at the things that they rank highest in the valuation. I do believe it helps me a little bit talking with them about it…” |
| Individual Health Professional Factors | Attitudes towards guidelines in general |  | Interviewee’s attitude towards P3P: Positive, receptive, neutral, or negative/hostile | Factors related to P3P in general – does the interviewee like/dislike P3P or has positive/negative opinions about P3P based on their experience | Opinions about prostate cancer guidelines (or other guidelines not related to P3P) | “Well, in theory, I like the idea of the concept to have something printed out to objectively capture the patient’s views.”  “I do think it [p3p] will also enable them to have a better baseline information when we meet and talk for the first time.” |
| Individual Health Professional Factors | Expected outcome |  | By implementing P3P, respondent expects to achieve the expected outcome | Include any expectations of P3P as a benefit to patients or providers | Expected outcomes related to procedures such as survival, potential morbidity issues | “Now with the P3P, that cuts out, one of those extra sessions. And that’s an advantage for me.” |
| Individual Health Professional Factors | Intention and motivation |  | Motivation to implement P3P. Do they want to continue implementing P3P? Has their motivation dampened? | Factors related to motivation—inertia, enthusiasm, drive, desire etc. | Do not include factors related to emotions (feelings) or nature of the behavior (habits) | “So, anytime you do something new in your practice, it’s hard to change what’s been working.” |
| Individual Health Professional Factors | Emotions |  | Emotions that facilitate or hinder adherence | Factors related to emotions – how they feel | Do not include factors related to how they think. | “No challenges from my perspective. Seems like there are no negative feedback from our office staff.” |
| Professional Behavior | Nature of the behavior |  | “This is what it is” type of sentiment. What do they do? Is it a habit? – characteristics of the behavior that are unique to the interviewee | How do they share biopsy results with patients? How do they operate their practices? How do they behave with their patients? | Behavior that has no impact on clinical operations (such as I make house calls) | “I am fearful that if I modify what I do with patients that I will forget something like I use, you know, statistical tables of chances of metastatic disease.” |
| Professional Behavior | Capacity to plan change |  | Interviewee’s capacity to plan for change in their workflow (bandwidth issues) | Capacity for change related to implementation, workflow changes | Factors that don’t pertain to individual physician/staff level capacity | “It does as I describe my flow. It would radically change my flow, which will make it little challenging for me.” |
| Professional Behavior | Self-monitoring or feedback |  | Interviewee’s ability to be reflective, self-monitoring of own’s behavior | Factors related to self-monitoring | Exclude factors related to performance, bonus etc. (monitoring by other entities) | “I basically try to hit on every topic and, you know, you modify it based on person’s state of health or their age or their level of fear, PSA, Gleason etc.” |
| Patient factors | Patient needs |  | Real or perceived needs and demands of patients. Tangible things. | Patient needs *x*. Patient doesn’t have *y* or patients can’t do it without clinic staff helping with *z*. | Patient preferences, motivation, or behavior. | “I do have patients who don’t have access to computers”  “The only time we have problems is when a person is not hooked up on the Internet.”  “We have to help some people do the survey” |
| Patient factors | Patient beliefs and knowledge |  | Patient knowledge gain, change in beliefs, ability to learn etc. | Beliefs that could hinder P3P implementation, knowledge gain as a result of P3P participation | Different from patient needs (beliefs + knowledge are not related to tangible things) | “Not only that, patients are able to interact more freely in the discussion because they come prepared (knowledge)  “… He felt that even on active surveillance, he’s worried that he might get metastatic disease.” |
| Patient factors | Patient preferences |  | Patients values related issues. Patient preferences. Intangible things. |  | Different from patient needs as preferences focus on intangible issues such as “mindset, “values” | “Some patients complain about it, they feel it’s intrusive.”  “I tell them the absolutely blank slate story for them to be able to fill in the blanks and try to make their own decisions.” (preferences) |
| Patient factors | Patient motivation |  | Patient does x because they anticipate y benefit (reward) or patient does x because they fear y penalty (loss aversion) | When patients do something because they are motivated, requested, asked by their providers | Motivation differs from needs and preferences because motivation is associated with gain/loss | “The positive aspect would be several patients have said that it kind of focused their mind on the task ahead and the task being they are going to make a decision.” |
| Patient factors | Patient behavior |  | Patient behaviors that impact P3P implementation | When patients behave in a way that hinders P3P. Such as patients refuse P3P, patients don’t adhere to physician recommendation to complete P3P etc. | Not related to motivation, preferences or beliefs. Behaviors that have direct impact on P3P implementation | “one of the problems is that information is overwhelming for most of them.”  “I have a pretty decent patient returns”  “…You have a person that just doesn’t want to be bothered with something like that.”  “In general, I don’t get any pushback over the phone.” |
| Professional Interactions | Team processes |  | Contributions of team members, how well the team functions | Skill set of team members, training, seamless hand-offs between physicians and nurses or other team members | Don’t include hierarchical structures, reporting that are not related to operational aspects of clinical processes | “I think the nurses have it down pretty good now.” |
| Determinants of Practice | Facilitators |  | Facilitating factors that contributed to **initial** successful implementation of P3P | These factors are specific to P3P that we weren’t able to code to the above codes | Responses that are directly associated with the “facilitator” question |  |
| Determinants of Practice | Barriers |  | Inhibiting factors that contribute to **initial** challenges to implementation of P3P | These factors are specific to P3P that we weren’t able to code to the above codes | Responses that are directly associated with the “barrier” question |  |
|  | Familiarity with P3P |  | Respondent has heard about P3P or attended P3P information session, or have read about P3P | Comparator site respondents were asked about familiarity/awareness of P3P | Responses that are directly associated with “awareness” question |  |
|  | P3P Efficacy |  | Whether the respondent mentioned about efficacy of P3P – i.e. “does it truly help patients” perspective |  | Responses that are associated or refer to “efficacy of the tool” type questions |  |
|  | Suggestions for MUSIC |  | Suggestions, advice or concerns respondent might have shared about MUSIC or staff affiliated with MUSIC |  |  |  |
|  | Tablet computers |  | Whether respondent has used MUSIC provided tablet computers |  |  |  |
